# A History of the Bristol Royal Infirmary

**Published:** 1917-12

**Authors:** 


### Reviews of Books.

**A History of the Bristol Royal Infirmary.** By G. Munro Smith, M.D., L.R.C.P. Lond., M.R.C.S., Hon. and Consulting Surgeon, Bristol Royal Infirmary, late Lieut.-Col. R.A.M.C. (T.). Pp. xiii. 507. Illustrated. Bristol: J. W. Arrowsmith Ltd. 1917.

"These to His Memory—since he held them dear."

This *magnum opus* of its lamented author was practically completed prior to the outbreak of the war, and prior to the long illness which culminated in the author's death on January 13th, 1917. He had corrected some of the proofs, but did not live to see the publication of the work which had occupied his thoughts for several years previously. He sought to give the history of the Infirmary from its foundation in 1736 to the building of the magnificent new surgical wing, which was opened by their Majesties King George V. and Queen Mary on June 28th, 1912. The splendid work which has since been accomplished there as the Second Southern General Hospital can only be recorded by some future historian after the termination of the most gigantic struggle in the world's history. These modern developments are largely due to the initiation and beneficence of the late President and Treasurer, Sir George White, Bart., who died on March 22nd, 1916. The author of this history, amongst his many other duties, military and civil, made it the chief business of his life to gather together the details and ensure the accuracy of the historic facts which are the foundation of the work. These were mainly compiled by an assiduous study of a row of bulky volumes, fourteen in number, labelled "Biographical Memoirs," which have been carefully preserved for many years in the Board Room of the Institution. They contain written accounts of Infirmary affairs—elections, lists of officers, notices of meetings, letters, newspaper cuttings, and historical memoranda, together with biographical histories of many of those who were connected with the Infirmary from the time of its foundation to the year 1842. They were collected by a man of literary tastes, a facile writer, and a born biographer, Richard Smith, who was Surgeon to the Infirmary from 1796 to 1843, and who founded the Infirmary Museum. Rarely has an institution found so faithful a recorder of antiquarian research and personal observation of the men and manners of his time, a record which the author states "has perhaps never been excelled except by James Boswell."

It was thought by the author that "if these memorials of by-gone times could be brought from their retreat into daylight, and be put into some kind of sequence and order, they could not fail to be of interest to many, not only as a history of a great charity, but as a means of looking with the eyes of a keen observer into the vivid life of an eventful epoch."

The writer further comments that "during the long period he has been working at the subject he often felt like the Editor of Professor Teufelsdröckh's Memoirs in *Sartor Resartus*. As he had to sort out and attempt to reduce to order six large paper bags containing miscellaneous masses of sheets, and oftener shreds and snips, treating of all imaginable things under the zodiac and above it, so an attempt has been made to fo m a continuous narrative out of the contents of these fourteen large volumes."

From this mass of material it is difficult to select special subjects for criticism or comment ; the book is one to be read at leisure and digested in detail. It is well printed in excellent type, and singularly free from errors of all kinds. The frontispiece is a very interesting portrait of Richard Smith, jun., and is from a pencil drawing by Branwhite, sen., hitherto unpublished ; the original was presented to the author by Mr. John E. Pritchard.

The matter is arranged in thirty-three chapters, with three appendices, and there are eighty-eight maps and illustrations, many of them being full-page portraits. But the story of the Bristol Royal Infirmary is not merely a question of how and when it was built, what endowment it had, and how much suffering it has relieved. These are worthy of permanent record, but are only a small part of its history. The history of the building is a history of the men who built it, it is they who have made it what it is.

Just as the Infirmary claims to be one of the earliest to be established in this country, it may also claim to have been the pioneer in the Sunday annual collections for the hospitals which are now almost universal, for we note that in the year 1750 the collections at churches and chapels amounted to about £345. In 1917 the amount collected was £3,159 7s. 0d., of which the Infirmary portion was £1,218.

The contrast between Then and Now is very striking. The general transformation and betterment of the Nursing Department, the abolition of the apothecary (page 127) and the barber-surgeon, the introduction of anæsthetics (chloroform was first used at the Infirmary in 1850), the enormous increase in the number and variety of surgical operations (in 1850 operations numbered 89, in 1912 they numbered 2,833, and every year they still increase), the frequent mention in the book of lectures on anatomy, and the devices of the body-snatchers, long anterior to the commencement of what has since been known as the Bristol Medical School and the Medical Faculty of the University of Bristol, are a few of the many questions which have been dealt with.

The introduction of antiseptic and aseptic methods to surgical work has been to a large extent responsible for the enormous increase in the number of operations during recent years, so many of them having been made possible where formerly they could not be attempted. It was mainly due to this fact that the building of the new surgical annexe became imperatively necessary, and was initiated by the lately-deceased President. The number of operations in 1915 was: major, 2451; minor, 701.

On the other hand, it is interesting to learn that from the beginning the privilege of voting by subscribers, and that of recommending patients has been strongly maintained, and persists as vigorously as ever, in spite of the motto "Charity Universal." It may well be claimed that the only qualification required should be that of being "sick and needy," and that the aid given should have no reference to charitable subscriptions or subscribers. Great vigilance was observed to prevent what was and is still named "hospital abuse," the visitors questioning every patient who came as to his inability to pay for medical attendance. This question has been frequently on the tapis during later years, and especially now that the Medical Insurance Schemes of the Government have to a great extent diminished the necessity for so great a crowd of out-patients who throng the rooms of that department.

Another point worthy of note is that of the virtue of punctuality insisted on (page 201), where we read "that from the foundation of the Bristol Infirmary it has been a point of honour with the surgical staff to leave any business or patient, however important, to obey an urgent summons to the 'House.' This, and punctuality at operations, have always been two wholesome traditions which have been scrupulously kept," and were strongly emphasised by the late President at the time of his appointment.

We read of some few of the differences, called by the author "squabbles," between various members of the medical and surgical staff, and between them and the Committee of Management, who in the year 1798 were denominated by one of the staff as "an oligarchical set of hornets." But since the time when it was arranged that two members of the staff in rotation should serve for a year on the Committee for the purpose of representing the staff on that body, these differences have ceased, and things have worked harmoniously for many years.

The talented writer, Mrs. Humphry Ward, in *Robert Elsmere,* describes a visit to an antiquarian library, where her hero had got " below the surface and was beginning to feel the joys of digging." He says, " I am only now beginning to dig for myself. Till now it has been work at second-hand. I have been getting a general survey of the ground as quickly as I could with the help of other men's labours." We can fancy that our author, having made this *hors d'œuvre* his *mètier,* must have derived immense satisfaction from his self-imposed duty, his digging having rescued a treasure from the old lumber ward, which had been simply a receptacle for the superfluous and useless. The work could not have been dreary when he remembered the words of Southey :—

" My days among the dead are passed ;
  Around me I behold,
Where'er these casual eyes are cast,
  The mighty minds of old ;
My never-failing friends are they,
  With whom I converse day by day.'

He must have found, with Elsmere, " that a passionate sense of the human problems which underlie all the dry and dusty detail of history and give it tone and colour, a passionate desire to rescue something more of human life from the drowning, submerging past, to realise for himself and others the solidarity and continuity of mankind's long struggle from the beginning until now," were well worth while, and he being dead yet speaks to us : " Go thou and do likewise."

All lovers of the old Infirmary will treasure the volume not only for its intrinsic interest, but as a memorial of its beloved author. [1]

